# Supplementary material for: Evaluation of Single-Molecule Sequencing Technologies for Structural Variant Detection in Two Swedish Human Genomes
Source: Genes (Basel). 2020 Nov 30;11(12):1444. doi: 10.3390/genes11121444 (PMC7760597; doi:10.3390/genes11121444)
Supplement: Supplementary file 1 [file genes-11-01444-s001.pdf]

Supplementary Materials

Table S1. Summary statistics for Oxford Nanopore Technology (ONT) sequencing. PromethION (beta) device was used for sequencing for all four flowcells. Yield and mean read length were calculated with NanoComp (github.com/wdecoster/nanocomp) [36] after basecalling. Key: Gb = Gigabases, bp = basepairs.

Library	Yield (Gb)	Estimated Genome Coverage	Maximum Length (bp)	Total Number of Reads	Read N50**	Total Number of Bases
OP001 (Swe1)	26.6	8.31x	838,077	4,208,279	26, 471	26,663,122,288
OP002* (Swe1)	77.9	24.34x	1,021,893	9,359,793	14, 311	77,920,585,462
OP003 (Swe2)	47.2	14.75x	679,796	6,805,305	20, 975	47,289,196,719
OP004* (Swe2)	57.18	17.87x	858,408	8,120,176	11, 944	57,185,367,919

**Read length of N50 is half of the total sequenced bases of reads equal or larger than the values mentioned.
*Sheared libraries.

Table S2. Read length and quality score for ONT (PromethION) sequencing. Metrics were obtained post-basecalling using NanoStat (github.com/wdecoster/nanostat) [36].

General summary	OP001 (Swe1)	* OP002 (Swe1)	OP003 (Swe2)	*OP004 (Swe2)
Mean read length	6,335	8,325	6,949	7,042
Mean read quality	6.9	7.8	7.5	7.6
Median read length	1,910	7,201	2,932	5,332
Median read quality	8.7	9.0	9.1	8.8

*Sheared libraries.

Table S3. Mapping ONT reads to GRCh38: Alignment statistics are based on raw data mapped to the reference assembly. Statistics were obtained with SAMtools version 1.9 (github.com/samtools/samtools) [39] after each alignment run. The resulting BAM files were later merged to a single file for each individual for structural variant (SV) calling.

Metrics	ONT (PromethION) Libraries			
	OP001 (Swe1)	OP002 (Swe1)	OP003 (Swe2)	OP004 (Swe2)
**Total Raw Sequences**	4,208,279	9,359,793	6,797,511*	8,120,176
**Mapped Reads (secondary alignments) [1]**	2,644,132 (816, 743)	7,172,690 (2,199,882)	4,771,239 (1,425,707)	6,120,418 (1,869,187)
**Mapped Reads ** (Forward & Reverse), Percentage**	70.12 (64.9 & 35.1)	82.15 (58.7 & 41.3)	76.38 (61.6 & 38.4)	80.79 (59.0 & 41.0)
**Bases Mapped [2]**	23,963,442,045	70,960,805,580	43,007,519,860	50,321,571,655
**Unmapped Reads**	1,564,147	2,187,103	2,026,272	1,999,758
**Runtime (CPU), hours**	2.8 (18.6)	7 (48.1)	5 (31.6)	7 (6.5)

* 7794 sequences were filtered out due to being invalid i.e. fastq entries being concatenated together.
[1] In a context of Minimap2 aligner, secondary alignments are alternative aligned reads that are tagged by “272” and “256” flags in SAM format. Primary alignments are the reads with longest alignments.
** Percentage representing a count of reference locations for mapped reads.
[2] Accurate estimate of mapped bases (based on CIGAR format).

Table S4. **Mapping PacBio reads to GRCh38**. Alignment statistics are based on raw data mapped to the reference assembly. Statistics were obtained with SAMtools after each alignment run.

SMRT Libraries	Total Sequences	Average Length (basepairs)	Maximum Length (basepairs)	Mapped Reads	Unmapped Reads	Runtime (CPU), hours
**Swe1**	26,395,733	8930	55,282	25,729,221	666,512	16.5 (133)
**Swe2**	26,780,995	8698	73,235	25,912,828	868,167	16.8 (135)

Table S5. Structural Variant (SV) Calling: ONT and PacBio Data. The events were called with Sniffles version 1.0.10 (github.com/fritzsedlazeck/Sniffles) [17]. Runs were performed with the following parameters: report_seq and --report_BND; where report_seq retains sequences for SVs detected as insertions and deletions, and report_BND enables detection of breaking-end events such as inversions.

SV Type	ONT Swe1	ONT Swe2	PacBio Swe1	PacBio Swe2
Insertions (INS)	9027	8608	12, 834	12, 615
Deletion (DEL)	8078	8133	9495	9531
Duplications (DUP)	209	163	374	330
Inversion (INV)	138	132	211	202
Translocation (TRA)	377	292	897	801
Duplication Insertion (DUP/INS)	1	0	4	6
Inverted Duplication (INVDUP)	1	1	13	761
Deletion/Inversion (DEL/INV)	4	4	9	10
**Total**	17,835	17,333	23, 837	24, 256
**Run Time (CPU), hours**	2.4 (6.1)	2.3 (6)	6.5 (15.9)	6.0 (15.5)

Table S6. Parameter testing for overlap detection. The results were obtained with SURVIVOR version 1.0.7 (github.com/fritzsedlazeck/Survivor) [42]. Limiting maximum allowed distance increases the number of merged SVs, while the number of SVs (present in Swedish genomes) that overlap with the truth set decreases.

**Maximum allowed distance between breakpoints**	**Merged SVs**<br>(ONT + PacBio + High Confidence Set from Chaisson *et al* [33])
5	104, 269
10	98, 435
50	82, 974
500	57, 415

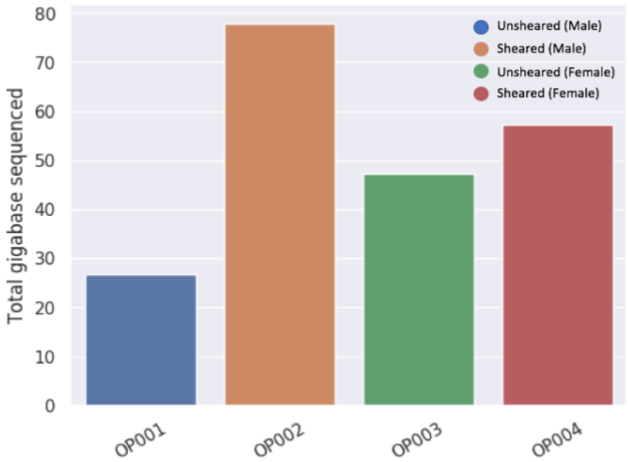

Figure S1. ONT Sequencing Yield. A bar plot displaying PromethION sequencing yield in Gb. Sheared libraries (OP002 and OP004) have a higher yield as compared to native DNA libraries (OP001 and OP003), and same pattern was observed in terms of coverage across all libraries. The plot was generated with NanoComp (github.com/wdecoster/NanoComp) [36].

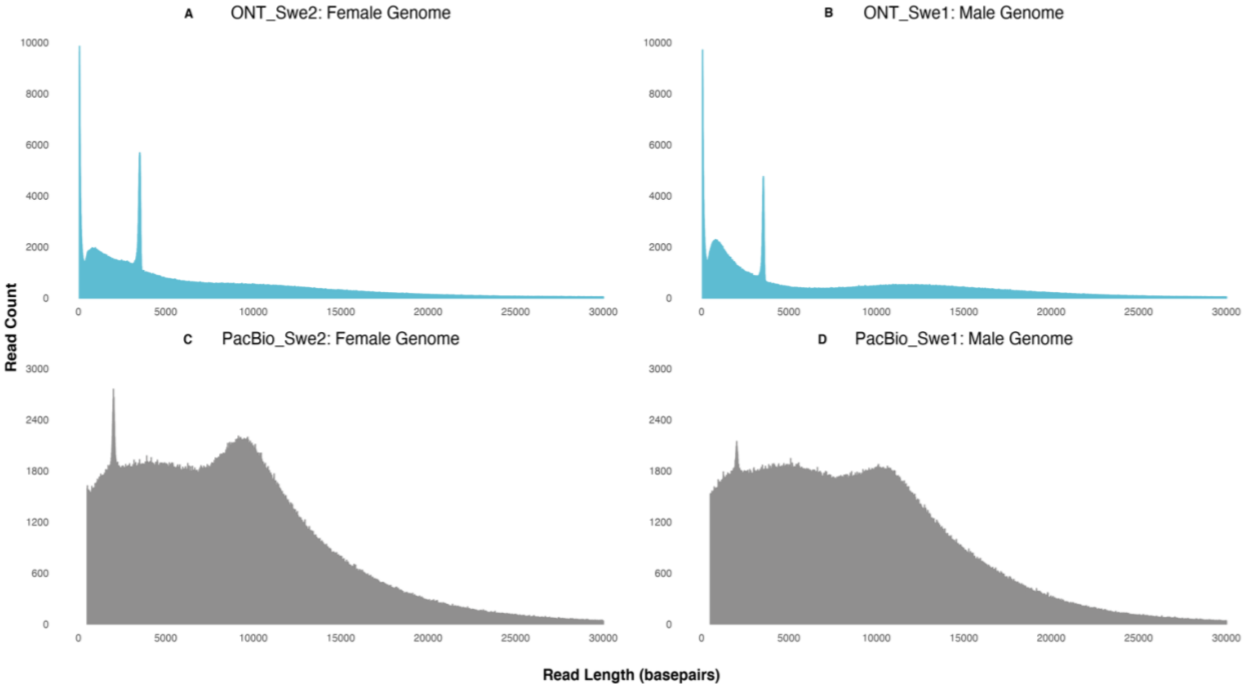

Figure S2. Distribution of Read Lengths. Read length distribution of ONT (top; A and B) and PacBio (bottom; C and D) sequencing runs. The data was collected from FASTQ files. For both x and y axes, the start of the interval is set to 0. For x-axis of all plots, the end interval is set to 30K basepairs (bp) with bins set to 5000 bp. For y-axis of ONT and PacBio plots; the end interval is set to 10K (bins: 2K) and 3K (bins: 600), respectively. The peaks in both plots denote control fragments. Plots were generated in R with ggplot2 (github.com/tidyverse/ggplot2) [43].
